# The relationship between violence history in patients with severe mental disorders and child abuse of their children

**DOI:** 10.7717/peerj.21028

**Published:** 2026-04-07

**Authors:** Meiling Wang, Lingfei Liu, Xiujun Liu, Wencai Chen

**Affiliations:** 1 Wuhan Hospital for Psychotherapy, Wuhan, Hubei, China; 2 Wuhan Mental Health Center, Wuhan, Hubei, China; 3 Department of Public Health, Jining Medical University, Jining, Shandong, China

**Keywords:** Children of parents with mental illness, Child abuse, Severe mental disorder, Violence history, Risk factor

## Abstract

**Background:**

Research on child abuse among children of parents with mental illness (COPMI) remains limited. This study aimed to investigate the prevalence of child abuse among COPMI and examined the association between a history of violence in parents with severe mental disorders (SMD) and the risk of child abuse in their households.

**Methods:**

A cross-sectional study was conducted among families of SMD patients registered at community health centers in Wuhan, China (2020). Demographic and clinical characteristics of participants and their parents were collected. Prevalence of child abuse was analyzed, and logistic regression model assessed associations between SMD patients’ violence history and child abuse.

**Results:**

Of 352 COPMI, 15.06% reported any abusive behavior in the past 6 months. Specifically, 14.20% experienced emotional abuse, 7.40% experienced physical abuse, and 7.10% experienced two or more specific abusive behaviors. Multivariable analysis revealed households with two mentally ill members, mental illness relapse, SMD duration ≤5 years, and violence history as significant child abuse risk factors.

**Conclusion:**

The 6-month prevalence of abuse (emotional or physical) among COPMI is notable. A clinical profile of the parent with SMD that includes a history of violence is associated with a significantly higher risk of abuse within the household. These findings inform potential adaptations of the China’s National Continuing Management and Intervention Program (‘686’ Program) for COPMI-focused prevention strategies.

## Introduction

Child abuse represents a pervasive social issue affecting both developed and developing nations (Finkelhor et al., 2015). The 2008 National Survey of Children’s Exposure to Violence (NatSCEV) reported that 10.1% of children and adolescents experienced maltreatment (including physical and emotional abuse, neglect, or family abduction) within the past year, with lifetime prevalence reaching 18.6% (Finkelhor et al., 2009). By 2014, these figures had risen to 15.2% and 24.9%, respectively (Finkelhor et al., 2015). A Turkish cross-sectional study found 22.8% of students had encountered violence during their lives (Oğuztürk et al., 2019). Similarly, a Chinese systematic review revealed physical abuse in 26.6% of minors, emotional abuse in 19.6%, sexual abuse in 8.7%, and neglect in 26.0%, contributing to an estimated 11.3 million Disability Adjusted Life Year (DALY) loss in 2010, a burden equivalent to diabetes mellitus and ischemic heart disease (Fang et al., 2015).

Victims of child abuse frequently develop hopelessness and distress, often manifesting as self-harm (Wong et al., 2011). Such trauma also correlates with lasting personality impairments that heighten vulnerability to subsequent victimization (Sansone, Chu & Wiederman, 2007). Substantial evidence links childhood abuse to adverse developmental outcomes, including reduced IQ and reading ability (Delaney-Black et al., 2002), impaired language processing (Cobos-Cali et al., 2018), and significant socioemotional and cognitive deficits (Margolin & Gordis, 2000). Beyond physical health consequences, abuse elevates risks for psychiatric conditions including depression, anxiety, and post-traumatic stress disorder (Gilbert et al., 2015; Jaffee, 2017; McKay et al., 2021). The enduring impacts prove particularly severe for children (Macmillan, 2001), with early victimization predicting adult criminal behavior and revictimization (Silverman, 1994) or the cycle of violence (Lewis, 1992).

While the absolute number of violent acts in the community attributable to severe mental disorders (SMD) like schizophrenia or bipolar disorder is very low (Joyal et al., 2007), they demonstrate higher violence rates than the general population (Labrum & Solomon, 2016). Family members represent over 50% of victims in incidents occurring within 2 weeks before hospitalization (Binder & McNeil, 1986), 22–36% of cases within the preceding year (Labrum & Solomon, 2015), and at least 40% since illness onset (Kageyama et al., 2015; Vaddadi, Gilleard & Fryer, 2002). Key risk factors include specific psychiatric diagnoses, early illness onset, frequent hospitalizations, treatment non-adherence, and disengagement from mental health services (Labrum & Solomon, 2016).

Despite growing recognition of child abuse’s high prevalence, its occurrence within families affected by SMD remains understudied. Understanding the determinants of such abuse is essential. This study investigates the association between child abuse and families with SMD members.

## Methods

### Design and participants

This cross-sectional study was conducted in Wuhan, China during April 2020. Participants were recruited from children whose parents had received regular community health care through China’s National Continuing Management and Intervention Program (‘686’ Program). Eligible SMD patients were first identified through the registry based on having children aged 9–17. Subsequently, one child per eligible family was invited to participate. The ‘686 Program’ is a national initiative in China, officially known as the “Central Government Support for the Local Management and Treatment of Severe Mental Disorders Program”. It provides an integrated hospital-community care, free essential medications, and systematic follow-up services for patients with severe mental disorders, encompassing the following six International Classification of Diseases 10th revision (ICD-10) conditions: schizophrenia (F20.-), bipolar disorder (F31.-), schizoaffective disorder (F25.-), persistent delusional disorder (F22.0), mental disorders due to epilepsy (coded with the corresponding F06.- subcategory, in conjunction with the underlying epilepsy code G40.-), and mental disorders in intellectual disability (coded with the corresponding F70–F79 code for the degree of disability, in conjunction with the relevant mental disorder code, typically from F00–F09). The ethics approval was obtained from Wuhan Mental Health Center (KY2020.02.05).

We conducted a multistage stratified random sampling across Wuhan’s 13 districts (seven urban, six rural). First, we randomly selected one urban (Hanyang) and one rural (Xinzhou) district. Next, we randomly chose one-third of Community Health Care Centers (CHCCs) in these districts and yielded 12 participating centers. At each center, we queried the Information Management System for Severe Mental Disorders to look for the registration information of patients with children aged 9 to 17. And the follow-up record of ‘686’ Program were also used to find appropriate participants. Children who, even with the help of their parents, still fail to understand the content of questionnaire will be excluded. Data collection occurred *via* online questionnaires, WeChat, or home visits by trained CHCC mental health workers, with written consent obtained from both parents and children.

The sample size was calculated by PASS software (version 15.0) according to the reported child abuse prevalence (5%), allowable error (0.025), α = 0.05, and dropout rate (10%), the minimum required sample size was 325. Ultimately, 90 participants from Hanyang district and 262 from Xinzhou district were enrolled.

The calculation formula is as follows: ${\rm n= (Z_{\alpha/2^{2}} * p * (1-p)) /\delta^2}.$

### Measurements

We recorded demographic characteristics for children (sex, age, school attendance status, residence) and their parents with SMD (sex, age, disease type, illness duration, number of psychiatric hospitalizations, violent behavior history). The SMD demographic data came from the CHCC, following the National Health Commission’s guidelines for managing severe mental disorders. For this study, key clinical variables were operationally defined as follows: ‘Relapse’ was defined as a documented exacerbation of psychiatric symptoms that required an increase in treatment intensity, emergency intervention, or re-hospitalization. ‘Hospitalization’ referred specifically to admissions to a psychiatric inpatient unit for acute stabilization or treatment of the mental disorder. Family-related factors included household poverty status, major medical conditions (including common chronic diseases such as hypertension, diabetes, asthma, gastric ulcers, and cancer) among co-residing relatives, and the number of mentally ill household members. Poverty status was verified using civil affairs department records.

A history of violent behavior was ascertained from the National Severe Mental Disorders Information Management System (NSMDIMS), with records reviewed from January 1, 2019. Violent behavior was defined based on the Risk Behavior Assessment scale, which classifies behavior into six levels (0–5). For this study, a positive violence history was defined as the documentation of any episode reaching Level 3 or above (Liu et al., 2018; Zhang et al., 2023). Individuals who had engaged in behaviors of Level 3 or higher were considered to have a positive history. This assessment is recorded in the NSMDIMS by CHCC mental health workers during each follow-up visit, with a minimum frequency of four times per year. In addition, police records of violent incidents involving the patients were reviewed as a supplementary source to ensure comprehensive ascertainment, particularly for severe cases. These records did not alter the primary classification based on the Risk Behavior Assessment scale; rather, any police-documented incident that met the above operational definition (Level 3–5) was used to corroborate and complement the clinical records from the NSMDIMS.

Child abuse assessment employed nine standardized questions adapted from the Chinese Adverse Childhood Experiences-International Questionnaire (Chen et al., 2022). The questionnaire assessed two types of exposure: five items pertained to witnessing abuse among household members, and four items pertained to being a direct victim of abuse. Specifically, for direct victimization, participants reported whether their father, mother, or other household members had exhibited four specific behaviors during the 6-month period (October 2019 to March 2020): verbal insults/humiliation/yelling, physical threats, mild physical aggression (pushing/grabbing/slapping/object throwing), or severe beating causing injuries. The first two behaviors constituted emotional abuse, while the latter two represented physical abuse. Responses were recorded on a 4-point Likert scale (0 = never, 1 = once, 2 = several times, 3 = many times). A participant was classified as having experienced child abuse if they reported exposure (score ≥1) to at least one of the four specific abusive behaviors. Accordingly, ‘Any abusive behavior reported’ was defined as exposure to at least one of these behaviors. ‘Two or more specific abusive behaviors’ was defined as exposure to two or more of them.

### Statistical analysis

Statistical analysis was performed using IBM SPSS software (version 21.0; SPSS Inc., Chicago, IL, USA). The characteristics of participants were summarized, and child abuse prevalence rates were computed. Categorical variables were compared using chi-square tests. Univariate and multivariate logistic regression analyses identified risk factors for child abuse, with odds ratios (ORs) and 95% confidence intervals (CI) quantifying association strength. Univariate results presented unadjusted ORs and *p*-values. Variables showing *p* < 0.10 in univariate analysis were retained for multivariate modeling. The final model was refined using a backward stepwise selection procedure with a removal criterion of *p* > 0.05. Predictors retained in the final model were selected based on both statistical significance and clinical relevance to the hypothesized risk mechanisms for child abuse in this population. Given the number of child abuse-positive events (*n* = 53), we aimed to ensure model stability by maintaining a parsimonious model. The final model includes four predictor variables, adhering to the common guideline of having at least 10 events per variable (EPV), which supports the robustness of the odds ratio estimates.

## Results

The study included 352 respondents with a mean age of 12.87 ± 2.47 years (range 9–17), comprising 185 boys (52.60%), 348 Han Chinese (98.86%), and 331 students (94.03%). Among 352 SMD patients, the mean age was 40.22 ± 5.19 years (range 29–53), with 104 males (29.55%), 281 schizophrenia diagnoses (79.83%), and 35 cases with violence history (9.94%). Tables 1 and 2 present complete demographic and clinical characteristics for both groups.

**Table 1:** Prevalence of child abuse in the 6-month period (*n* = 352).

Traumatic experiences	*n* (%)
**Specific abusive behaviors reported**	
1. Abuse or ridicule	50/352 (14.20%)
2. Being threatened	13/352 (3.69%)
3. Being pushed, grabbed or thrown	24/352 (6.82%)
4. Severe beatings	15/352 (4.26%)
**Derived abuse categories**	
**Any abusive behavior reported (≥1 behavior)**	53/352 (15.06%)
Emotional abuse (behavior 1 or 2)	50/352 (14.20%)
Physical abuse (behavior 3 or 4)	26/352 (7.40%)
**Two or more specific abusive behaviors**	25/352 (7.10%)

**Note:**

The survey was conducted to assess the 6-month prevalence (October 2019 to March 2020) of emotional and physical abuse experienced by children of parents with severe mental illness (COPMI). Emotional abuse was defined as experiencing either abuse/ridicule or threats. In this sample, all children who reported threats (*n* = 13) also reported abuse/ridicule; thus, the total count for emotional abuse (*n* = 50) is identical to the count for abuse/ridicule. Two or more specific abusive behaviors refers to experiencing two or more of the four specific behaviors assessed (ridicule, threats, mild physical aggression, severe beating).

**Table 2:** Characteristics of COPMI exposed to child abuse (*n* = 352).

Characteristics	Category	Child abuse: no (*n* = 299) *n* (%)	Child abuse: yes (*n* = 53) *n* (%)	χ^2^	*p*	Odds ratio (95% CI)
**Child factors**						
Sex	Boy	154 (51.51)	31 (58.49)	0.881	0.348	1
	Girl	145 (48.49)	22 (41.51)			0.75 [0.42–1.36]
Age (years)	9–11	100 (33.44)	19 (35.85)	0.164	0.921	1
	12–14	113 (37.79)	20 (37.74)			0.93 [0.47–1.84]
	15–17	86 (28.76)	14 (26.42)			0.86 [0.41–1.81]
Currently in school	Yes	283 (94.65)	48 (90.57)	1.338	0.247	1
	No	16 (5.35)	5 (9.43)			1.84 [0.65–5.26]
**Household & socioeconomic factors**						
Residence place	Rural	228 (76.25)	34 (64.15)	3.465	0.063	1
	Urban	71 (23.75)	19 (35.85)			1.80 [0.96–3.34]
Poverty status	Non-poverty	51 (17.06)	9 (16.98)	0.000	0.989	1
	Poverty	248 (82.94)	44 (83.02)			1.01 [0.46–2.19]
**Family clinical factors**						
Number of mentally ill household members	One	279 (93.31)	44 (83.02)	6.398	0.012	1
	Two	20 (6.69)	9 (16.98)			2.85 [1.22–6.67]
Presence of physical health problems in family members	No	284 (94.98)	45 (84.91)	7.487	0.006	1
	Yes	15 (5.02)	8 (15.09)			3.37 [1.35–8.40]
Relapse of mental illness in family members	No	272 (90.97)	34 (64.15)	28.503	<0.001	1
	Yes	27 (9.03)	19 (35.85)			5.63 [2.83–11.19]
**SMD patient factors**						
Sex	Male	88 (29.43)	16 (30.19)	0.012	0.911	1
	Female	211 (70.57)	37 (69.81)			1.04 [0.55–1.96]
Age (years)	~40	142 (47.49)	22 (41.51)	0.863	0.649	1
	40–50	141 (47.16)	27 (50.94)			1.24 [0.67–2.27]
	50~	16 (5.35)	4 (7.55)			1.61 [0.49–5.27]
Duration of illness (years)	>5	273 (91.3)	39 (73.58)	12.648	<0.001	1
	≤5	26 (8.70)	14 (26.42)			3.77 [1.81–7.83]
Diagnosis	Schizophrenia	237 (79.26)	44 (83.02)	0.394	0.530	1
	Others	62 (20.74)	9 (16.98)			0.78 [0.36–1.69]
Education level	Illiterate or primary school	132 (44.15)	23 (43.4)	0.183	0.913	1
	Junior middle school	122 (40.80)	23 (43.4)			1.08 [0.58–2.03]
	Senior high school and above	45 (15.05)	7 (13.21)			0.89 [0.36–2.22]
Hospitalization	Once	145 (48.49)	21 (39.62)	3.067	0.216	1
	Twice	88 (29.43)	22 (41.51)			1.73 [0.90–3.32]
	More than twice	66 (22.07)	10 (18.87)			1.05 [0.47–2.34]
Violence history	No	275 (91.97)	42 (79.25)	8.145	0.004	1
	Yes	24 (8.03)	11 (20.75)			3.00 [1.37–6.57]

Note:

Other types of severe mental disorders including schizoaffective disorder, persistent delusional disorder, mental disorders due to epilepsy, and mental disorders in intellectual disability. The odds ratios (OR) presented are unadjusted estimates derived from univariate logistic regression analyses. Adjusted ORs from the multivariable model are presented in Table 4.

Fifty-three respondents (15.06%) reported experiencing child abuse in the 6-month period. Emotional abuse (defined as exposure to abuse/ridicule or threats) was reported by 50 participants (14.20%). Notably, all cases involving threats were also reported as abuse/ridicule. Physical abuse was reported by 26 participants (7.40%), and 25 participants (7.10%) experienced two or more specific abusive behaviors. Specific incidents included 50 cases (14.20%) of abuse or ridicule, 13 (3.69%) involving threats, 24 (6.82%) involving physical handling (pushing, grabbing, or throwing), and 15 (4.26%) involving severe beating (Table 1).

Table 3 presents the distribution of violence history among patients and the reported child abuse across different SMD diagnostic groups. The sample with patients diagnosed with schizophrenia constituting the vast majority (*n* = 281, 79.8%). The other diagnostic groups were represented by considerably smaller samples (ranging from *n* = 2 to *n* = 32). Within the largest schizophrenia group, 11.0% of patients had a violence history, and 15.7% of their children reported abuse. A numerically high rate of abuse was reported in the bipolar disorder group (26.3%, 5/19). It should be noted that within the very small subset of patients in this group who had a violence history (*n* = 2), no abuse was reported.

**Table 3:** The distribution of child abuse and violence in different SMD groups (*n* = 352).

Types of SMD	N1	History of Violence	Emotional abuse	Physical abuse	Any abusive behavior reported
		(n, %)	(*n*, %)	(*n*, %)	(*n*, %)
Schizophrenia	281	31 (11.39)	42 (14.95)	22 (7.83)	44 (15.66)
Bipolar disorder	19	2 (10.53)	5 (26.32)	1 (5.26)	5 (26.32)
Schizoaffective disorder	4	0 (0)	1 (25.00)	1 (25.00)	1 (25.00)
Persistent delusional disorder	2	0 (0)	0 (0)	0 (0)	0 (0)
Mental disorders due to epilepsy	14	1 (7.14)	0 (0)	0 (0)	0 (0)
Mental disorders in intellectual disability	32	1 (3.13)	2 (6.25)	2 (6.25)	3 (9.38)

**Note:**

The distribution of child abuse and violence history across diagnostic groups is presented. Due to very small sample sizes in non-schizophrenia groups, these data are for descriptive purposes only; statistical comparisons are not appropriate.

Univariate analysis (Table 2) showed significant associations between child abuse and households with two mentally ill members, physical health problems, mental illness relapse, duration of illness, and violence history (*p* < 0.05). All variables with *p* < 0.1 in the univariate analysis were entered into the multivariate model. Multivariate analysis (Table 4) showed that the risk of children of parents with mental illness (COPMI) was significantly associated with households having two (*vs* one) mentally ill members (OR: 3.24), mental illness relapse (OR: 4.84), SMD duration ≤5 years (OR: 3.17), and SMD violence history (OR: 2.86).

**Table 4:** Multivariable risk factors for child abuse among COPMI.

Predictor	Risk	Reference	B	SE	Wald	*p*	Odds ratio (95% CI)
Number of mentally ill household members	Two	One	1.174	0.474	6.129	0.013	3.24 [1.28–8.20]
Relapse of mental illness	Yes	No	1.578	0.369	18.321	<0.001	4.84 [2.35–9.98]
Duration of SMD	≤5	>5	1.152	0.400	8.281	0.004	3.17 [1.44–6.94]
Violence history in SMD patient	Yes	No	1.049	0.437	5.767	0.016	2.86 [1.21–6.73]

## Discussion

To our knowledge, this study represents the first investigation of child abuse among COPMI in China. Evidence indicates that individuals with psychiatric disorders exhibit higher rates of family violence than the general population (Labrum & Solomon, 2015). In our research, we hypothesized that COPMI may face elevated abuse risks in families where parents with SMD have violent histories. Our findings demonstrate that children living in households where the SMD patient had a history of violence face a 2.86-fold greater risk of abuse within 6 months compared to those with non-violent SMD parents. Additional risk factors included households with two mentally ill members, recent mental illness relapse, SMD duration ≤5 years, and a history of violence in the SMD patient.

Childhood abuse exposure appears prevalent across populations. In our study, 15.06% of COPMI reported abuse within a 6-month period, including 14.20% emotional and 7.40% physical abuse. These rates are notable as they were observed in families with SMD during the COVID-19 pandemic, a period when social isolation and elevated stress levels may have increased the risk of child abuse (Griffith, 2022; Pereda & Diaz-Faes, 2020). Contrary to some expectations, the prevalence we found was lower than global estimates of childhood domestic violence (Whitten et al., 2024), previous Chinese maltreatment studies (Fang et al., 2015), and caregivers report (Labrum & Solomon, 2016). One possible explanation is the relatively short assessment window in our study. This is illustrated by the higher, lifetime exposure rate of 23.5% reported in the long-term New Zealand Birth Cohort Study (Rouland & Vaithianathan, 2018). In our sample, the reported prevalence of abuse was numerically higher in urban areas, where stricter isolation measures were implemented than in rural areas, although this difference was not statistically significant. This observation suggests that contextual factors may be at play and warrant investigation in future studies with larger, adequately powered samples. Despite the potentially unique context, the findings confirm that COPMI face a risk of abuse, underscoring the need for ongoing vigilance and intervention.

The literature consistently identifies SMD as major risk factors for violent behavior, particularly among affected patients (Labrum et al., 2021). Relapse of mental illness and prior violent acts emerge as robust predictors of aggression, with studies reporting violent incidents in 45% and aggressive behaviors in 33% of patients during psychiatric hospitalizations (Colasanti et al., 2008). Fazel et al.’s (2017) work specifically established violent crime history as the strongest indicator of short-term violent recidivism in SMD populations. This pattern extends to domestic settings, as Italian research found SMD patients with violent histories committed significantly more lifetime domestic violence than their nonviolent counterparts (Barlati et al., 2019). Our findings extend this established relationship to the family context, demonstrating that illness relapse and a history of violence in the SMD patient are associated with a substantially increased risk of abuse exposure for COPMI.

Moreover, we unexpectedly found that 8.24% of families contained two mentally ill members, substantially increasing COPMI’s abuse risk relative to single-patient households. No prior study has documented multiple psychiatric patients cohabiting within families. These results demonstrate that multi-patient households impose greater caregiver burdens while simultaneously elevating COPMI’s vulnerability to abuse. Additionally, our study found that child abuse risk peaks within the first five years after an SMD diagnosis, consistent with reports showing elevated violence risk during this time frame following schizophrenia or bipolar disorder diagnoses (Whiting, Lichtenstein & Fazel, 2021). These findings underscore the need for targeted early intervention in clinical settings to reduce violence risk during this critical window.

Our analysis of diagnostic-specific risks was constrained by the highly uneven sample distribution, which reflects the real-world caseload in China’s community mental health management (Wang et al., 2023). Given the very small subsamples, especially of patients with a violence history in non-schizophrenia groups, meaningful statistical comparison across diagnoses was not feasible. Therefore, the numerical differences in abuse prevalence shown in Table 3 are strictly descriptive and should not be used to draw conclusions about differential risk between diagnostic categories. This practical reality suggests that family-level clinical risk factors (*e.g*., parental violence history, illness relapse) may be more salient for child outcomes than the specific parental diagnosis. In contrast to some general population studies which find associations between child abuse and factors like child’s sex (Whitten et al., 2024) or family socioeconomic status (Bowen, 2015; Fong, Hawes & Allen, 2019), our analysis within this specific COPMI cohort revealed no such significant associations. It is noteworthy that some factors significantly in univariate analysis (*e.g*., physical health problems) did not retain significance in the multivariable model, suggesting their effect may be mediated or confounded by the more proximal clinical risk factors retained in the final model, such as mental illness relapse and violence history. Thus, the distinctive demographic and clinical context of COPMI, characterized by SMD, may attenuate or alter the role of more common risk factors, highlighting the critical need for targeted risk assessments in this population.

## Conclusions

Although limited by its sample size, this study provides initial evidence on emotional and physical abuse among COPMI in China, where comprehensive data in this area are currently scarce. Our analysis revealed a 6-month prevalence of child abuse of 15.06%, and identified several clinical and family-related factors associated with an increased risk of child abuse. These include a family environment where the SMD patient has a history of violence, shorter illness duration (≤5 years), relapse of mental illness, and the presence of two (*vs* one) mentally ill household members. Future research with larger, prospective cohorts is warranted to validate these findings and explore other forms of abuse. Nonetheless, our results suggest that public health initiatives, such as China’s ‘686’ Program, could be leveraged for the early identification of COPMI at risk and the implementation of targeted preventive strategies.

### Limitations

First, a limitation to generalizability is that data collection occurred during the COVID-19 pandemic (April 2020), a period of unique societal stress. Our results should be generalized with caution to non-pandemic periods or different cultural and policy contexts. Second, and specific to our study design, the assessment timeframes for the exposure (patient violence history) and the outcome (child abuse) were not identical. Patient violence history was ascertained from records dating back to January 1, 2019, whereas child abuse was reported for the 6 months preceding the survey (October 2019 to March 2020). This partial temporal overlap means that while we can establish a significant association, we cannot with certainty determine the exact temporal sequence or assert causality between the recorded violent behavior and the reported abuse incidents. Furthermore, while our results identified an association between child abuse and households affected by SMD, the study could not determine the perpetrators of abuse, limiting conclusions about violence specifically attributable to individuals with SMD. Additionally, the research relied on retrospective self-reports to measure child abuse exposure, which, though valuable for capturing unreported cases, remain vulnerable to recall and response biases. Finally, our child abuse assessment omitted emotional neglect, physical neglect, and sexual abuse compared to standard scales, possibly leading to an underestimation of abuse prevalence.

## Supplemental Information

10.7717/peerj.21028/supp-1 Supplemental Information 1 STROBE Statement.

10.7717/peerj.21028/supp-2 Supplemental Information 2 Risk Behavior Assessment scale.

10.7717/peerj.21028/supp-3 Supplemental Information 3 Nine standardized questions to assess abuse.

10.7717/peerj.21028/supp-4 Supplemental Information 4 Data.

10.7717/peerj.21028/supp-5 Supplemental Information 5 Variable code.

## Additional Information and Declarations

### Data Availability

The following information was supplied regarding data availability:

The raw data are available in the Supplemental File.

## References

[ref-1] Barlati S, Stefana A, Bartoli F, Bianconi G, Bulgari V, Candini V, Carrà G, Cavalera C, Clerici M, Cricelli M, Ferla MT, Ferrari C, Iozzino L, Macis A, Vita A, de Girolamo G (2019). Violence risk and mental disorders (VIORMED-2): a prospective multicenter study in Italy. PLOS ONE.

[ref-2] Binder RL, McNeil DE (1986). Victims and families of violent psychiatric patients. The Bulletin of the American Academy of Psychiatry and the Law.

[ref-3] Bowen E (2015). The impact of intimate partner violence on preschool children’s peer problems: an analysis of risk and protective factors. Child Abuse & Neglect.

[ref-4] Chen W, Yu Z, Wang L, Gross D (2022). Examining childhood adversities in Chinese health science students using the simplified Chinese version of the adverse childhood experiences-international questionnaire (SC-ACE-IQ). Adversity and Resilience Science.

[ref-5] Cobos-Cali M, Ladera V, Perea MV, García R (2018). Language disorders in victims of domestic violence in children’s homes. Child Abuse & Neglect.

[ref-6] Colasanti A, Natoli A, Moliterno D, Rossattini M, De Gaspari IF, Mauri MC (2008). Psychiatric diagnosis and aggression before acute hospitalisation. European Psychiatry.

[ref-7] Delaney-Black V, Covington C, Ondersma SJ, Nordstrom-Klee B, Templin T, Ager J, Janisse J, Sokol RJ (2002). Violence exposure, trauma, and IQ and/or reading deficits among urban children. Archives of Pediatrics & Adolescent Medicine.

[ref-8] Fang X, Fry DA, Ji K, Finkelhor D, Chen J, Lannen P, Dunne MP (2015). The burden of child maltreatment in China: a systematic review. Bulletin of the World Health Organization.

[ref-9] Fazel S, Wolf A, Larsson H, Lichtenstein P, Mallett S, Fanshawe TR (2017). Identification of low risk of violent crime in severe mental illness with a clinical prediction tool (Oxford Mental Illness and Violence tool [OxMIV]): a derivation and validation study. The Lancet Psychiatry.

[ref-10] Finkelhor D, Turner H, Ormrod R, Hamby S, Kracke K (2009). Children’s exposure to violence: a comprehensive national survey. Juvenile Justice Bulletin.

[ref-11] Finkelhor D, Turner H, Shattuck A, Hamby S (2015). Prevalence of childhood exposure to violence, crime, and abuse: results from the national survey of children’s exposure to violence. JAMA Pediatrics.

[ref-12] Fong VC, Hawes D, Allen JL (2019). A systematic review of risk and protective factors for externalizing problems in children exposed to intimate partner violence. Trauma, Violence, & Abuse.

[ref-13] Gilbert LK, Breiding MJ, Merrick MT, Thompson WW, Ford DC, Dhingra SS, Parks SE (2015). Childhood adversity and adult chronic disease: an update from ten states and the District of Columbia. American Journal of Preventive Medicine.

[ref-14] Griffith AK (2022). Parental burnout and child maltreatment during the COVID-19 pandemic. Journal of Family Violence.

[ref-15] Jaffee SR (2017). Child maltreatment and risk for psychopathology in childhood and adulthood. Annual Review of Clinical Psychology.

[ref-16] Joyal CC, Dubreucq JL, Gendron C, Millaud F (2007). Major mental disorders and violence: a critical update. Current Psychiatry Reviews.

[ref-17] Kageyama M, Yokoyama K, Nagata S, Kita S, Nakamura Y, Kobayashi S, Solomon P (2015). Rate of family violence among patients with schizophrenia in Japan. Asia Pacific Journal of Public Health.

[ref-18] Labrum T, Solomon PL (2016). Factors associated with family violence by persons with psychiatric disorders. Psychiatry Research.

[ref-19] Labrum T, Solomon PL (2015). Rates of victimization of violence committed by relatives with psychiatric disorders. Journal of Interpersonal Violence.

[ref-20] Labrum T, Zingman MA, Nossel I, Dixon L (2021). Violence by persons with serious mental illness toward family caregivers and other relatives: a review. Harvard Review of Psychiatry.

[ref-21] Lewis DO (1992). From abuse to violence: psychophysiological consequences of maltreatment. Journal of the American Academy of Child & Adolescent Psychiatry.

[ref-22] Liu Y, Liu X, Wen H, Wang D, Yang X, Tang W, Li Y, Zhang T, Yang M (2018). Risk behavior in patients with severe mental disorders: a prospective study of 121,830 patients managed in rural households of western China. BMC Psychiatry.

[ref-23] Macmillan R (2001). Violence and the life course: the consequences of victimization for personal and social development. Annual Review of Sociology.

[ref-24] Margolin G, Gordis EB (2000). The effects of family and community violence on children. Annual Review of Psychology.

[ref-25] McKay MT, Cannon M, Chambers D, Conroy RM, Coughlan H, Dodd P, Healy C, O’Donnell L, Clarke MC (2021). Childhood trauma and adult mental disorder: a systematic review and meta-analysis of longitudinal cohort studies. Acta Psychiatrica Scandinavica.

[ref-26] Oğuztürk Ö, Demir N, Bülbül S, Türkel Y, Ünlü E (2019). Exposure to domestic violence and its effects on adolescents: a survey among Turkish students. Journal of Child and Adolescent Psychiatric Nursing.

[ref-27] Pereda N, Diaz-Faes DA (2020). Family violence against children in the wake of COVID-19 pandemic: a review of current perspectives and risk factors. Child and Adolescent Psychiatry and Mental Health.

[ref-31] Rouland B, Vaithianathan R (2018). Cumulative prevalence of maltreatment among New Zealand children, 1998–2015. American Journal of Public Health.

[ref-28] Sansone RA, Chu J, Wiederman MW (2007). Self-inflicted bodily harm among victims of intimate-partner violence. Clinical Psychology & Psychotherapy.

[ref-29] Silverman RBRA (1994). Review: crime in the making: pathways and turning points through life. Canadian Journal of Sociology.

[ref-30] Vaddadi KS, Gilleard C, Fryer H (2002). Abuse of carers by relatives with severe mental illness. International Journal of Social Psychiatry.

[ref-32] Wang J, Su J, Sun X, Hou X, Chen X, Zhao Y, Yu H, Song H, Xu G, Zhou L (2023). Characteristics associated with registration and management of the 686 Program among schizophrenia patients in China: a cross-sectional study. Asian Journal of Psychiatry.

[ref-33] Whiting D, Lichtenstein P, Fazel S (2021). Violence and mental disorders: a structured review of associations by individual diagnoses, risk factors, and risk assessment. The Lancet Psychiatry.

[ref-34] Whitten T, Tzoumakis S, Green MJ, Dean K (2024). Global prevalence of childhood exposure to physical violence within domestic and family relationships in the general population: a systematic review and proportional meta-analysis. Trauma, Violence, & Abuse.

[ref-35] Wong SPY, Wang C, Meng M, Phillips MR (2011). Understanding self-harm in victims of intimate partner violence: a qualitative analysis of calls made by victims to a crisis hotline in China. Violence Against Women.

[ref-36] Zhang L, Qi X, Wen L, Hu X, Mao H, Pan X, Zhang X, Fang X (2023). Identifying risk factors to predict violent behaviour in community patients with severe mental disorders: a retrospective study of 5277 patients in China. Asian Journal of Psychiatry.

