# Supplementary material for: The relationship between violence history in patients with severe mental disorders and child abuse of their children
Source: PeerJ. 2026 Apr 7;14:e21028. doi: 10.7717/peerj.21028 (PMC13068010; doi:10.7717/peerj.21028)
Supplement: Supplemental Information 1 [file peerj-14-21028-s001.docx]

STROBE Statement—checklist of items that should be included in reports of observational studies

|  | Item No. | Recommendation | Page  No. | Relevant text from manuscript |
| --- | --- | --- | --- | --- |
| **Title and abstract** | 1 | (*a*) Indicate the study’s design with a commonly used term in the title or the abstract | 1 | A cross-sectional study was conducted with participants from families of SMD patients registered in community health centers in Wuhan, China(2020). |
|  |  | (*b*) Provide in the abstract an informative and balanced summary of what was done and what was found | 1 | This study investigates the prevalence of child abuse among COPMI and assesses whether severe mental disorders(SMD) increase the risk of child abuse.  The proportion of COPMI exposed to child abuse is relatively low but significantly linked to SMD. |
| Introduction | | | |  |
| Background/rationale | 2 | Explain the scientific background and rationale for the investigation being reported | 3 | Child abuse is a serious social problem prevalent in both developed and developing countries |
| Objectives | 3 | State specific objectives, including any prespecified hypotheses | 3 | Identifying the factors associated with the child abuse is crucial. Therefore, the objective of this study was to examine the relationship between child abuse and individuals with SMD. |
| Methods | | | |  |
| Study design | 4 | Present key elements of study design early in the paper | 4 | This cross-sectional study was conducted in April 2020 in Wuhan, China. |
| Setting | 5 | Describe the setting, locations, and relevant dates, including periods of recruitment, exposure, follow-up, and data collection | 4-5 | The parent of participants were diagnosed with schizophrenia, bipolar disorder, mental retardation with psychotic symptoms, or schizophrenia-like psychosis in epilepsy according to the ICD-10 criteria and were receiving regular community health care through the National Continuing Management and Intervention Program (‘686’ Program). |
| Participants | 6 | (*a*) *Cohort study*—Give the eligibility criteria, and the sources and methods of selection of participants. Describe methods of follow-up  *Case-control study*—Give the eligibility criteria, and the sources and methods of case ascertainment and control selection. Give the rationale for the choice of cases and controls  *Cross-sectional study*—Give the eligibility criteria, and the sources and methods of selection of participants | 5 | The inclusion criteria were as follows: 1.patients were registered at the CHCC. 2. Participants were aged 9 to 17 who had lived with their SMD parents for more than six months prior to the survey. Children with any serious disease prevented them from understanding the questionnaire with the help of their parents and investigators, as well as their gurdians or children refused to participate, were excluded. |
|  |  | (*b*) *Cohort study*—For matched studies, give matching criteria and number of exposed and unexposed  *Case-control study*—For matched studies, give matching criteria and the number of controls per case |  |  |
| Variables | 7 | Clearly define all outcomes, exposures, predictors, potential confounders, and effect modifiers. Give diagnostic criteria, if applicable | 6-7 | The Risk Behavior Assessment scale categorized violent behavior into six levels: Level 0-5. Level 3 and above indicate significant risk to hurt others^[22, 23]^, and were considered to have a history of violent behavior in this study. |
| Data sources/ measurement | 8* | For each variable of interest, give sources of data and details of methods of assessment (measurement). Describe comparability of assessment methods if there is more than one group | *6* | Demographic characteristics of children(sex, age, school attendance status, residential place) and SMD parents(sex, age, disease type, illness duration, number of psychiatric hospitalizations, history of violent behavior) were recorded. |
| Bias | 9 | Describe any efforts to address potential sources of bias |  |  |
| Study size | 10 | Explain how the study size was arrived at | 5-6 | The minimum sample size was calculated using the formula for cross-sectional studies. |

Continued on next page

| Quantitative variables | 11 | Explain how quantitative variables were handled in the analyses. If applicable, describe which groupings were chosen and why | 7 | In our study, no child abuse was defined as the absence of emotional and physical abuse, and child abuse was defined as having been exposed to at least one form of abuse. |
| --- | --- | --- | --- | --- |
| Statistical methods | 12 | (*a*) Describe all statistical methods, including those used to control for confounding | 7 | multivariate logistic regression were performed to reveal the risk factors for child abuse. |
|  |  | (*b*) Describe any methods used to examine subgroups and interactions |  |  |
|  |  | (*c*) Explain how missing data were addressed |  |  |
|  |  | (*d*) *Cohort study*—If applicable, explain how loss to follow-up was addressed  *Case-control study*—If applicable, explain how matching of cases and controls was addressed  *Cross-sectional study*—If applicable, describe analytical methods taking account of sampling strategy | 5 | A multistaged stratified random sampling method was used. |
|  |  | (*e*) Describe any sensitivity analyses |  |  |
| Results | | | | |
| Participants | 13* | (a) Report numbers of individuals at each stage of study—eg numbers potentially eligible, examined for eligibility, confirmed eligible, included in the study, completing follow-up, and analysed | 6 | The qualification rate was 95.39% (352/369). |
|  |  | (b) Give reasons for non-participation at each stage |  | 17 cases were excluded because their age was 18; |
|  |  | (c) Consider use of a flow diagram |  |  |
| Descriptive data | 14* | (a) Give characteristics of study participants (eg demographic, clinical, social) and information on exposures and potential confounders | 7-8 | The average age of the 352 respondents was 12.87±2.47 years (range 9–17), and 185(52.60%) were boys, 348 (98.86%) were ethnic Han, and 331 (94.03%) were students. |
|  |  | (b) Indicate number of participants with missing data for each variable of interest |  |  |
|  |  | (c) *Cohort study*—Summarise follow-up time (eg, average and total amount) |  |  |
| Outcome data | 15* | *Cohort study*—Report numbers of outcome events or summary measures over time |  |  |
|  |  | *Case-control study—*Report numbers in each exposure category, or summary measures of exposure |  |  |
|  |  | *Cross-sectional study—*Report numbers of outcome events or summary measures | *6* | 352 children who met the requirements participated in the study. The average age of the 352 respondents was 12.87±2.47 years (range 9–17). |
| Main results | 16 | (*a*) Give unadjusted estimates and, if applicable, confounder-adjusted estimates and their precision (eg, 95% confidence interval). Make clear which confounders were adjusted for and why they were included | 8 | Table 2 compares the characteristics between child abuse and non-child abuse. Univariate analysis identified households with multiple mentally ill individuals, |
|  |  | (*b*) Report category boundaries when continuous variables were categorized |  |  |
|  |  | (*c*) If relevant, consider translating estimates of relative risk into absolute risk for a meaningful time period |  |  |

Continued on next page

| Other analyses | 17 | Report other analyses done—eg analyses of subgroups and interactions, and sensitivity analyses |  |  |
| --- | --- | --- | --- | --- |
| Discussion | | | | |
| Key results | 18 | Summarise key results with reference to study objectives | 9 | Our findings reveal that children whose parents with SMD had a history of violence have a 2.86 times higher risk of being subjected to child abuse within six months compared to children whose SMD parents had no history of violence. |
| Limitations | 19 | Discuss limitations of the study, taking into account sources of potential bias or imprecision. Discuss both direction and magnitude of any potential bias | 12 | First, although our results revealed a relationship between child abuse and SMD, but the study did not identify the perpetrators of child abuse, limiting the ability to attribute violence specifically to individuals with SMD. |
| Interpretation | 20 | Give a cautious overall interpretation of results considering objectives, limitations, multiplicity of analyses, results from similar studies, and other relevant evidence | 12 | Although the current study is based on a small sample of participants, we found that the incidence of violence at COPMI over the past six months was 15.06%. |
| Generalisability | 21 | Discuss the generalisability (external validity) of the study results |  |  |
| Other information | |  | | |
| Funding | 22 | Give the source of funding and the role of the funders for the present study and, if applicable, for the original study on which the present article is based |  |  |

*Give information separately for cases and controls in case-control studies and, if applicable, for exposed and unexposed groups in cohort and cross-sectional studies.

**Note:** An Explanation and Elaboration article discusses each checklist item and gives methodological background and published examples of transparent reporting. The STROBE checklist is best used in conjunction with this article (freely available on the Web sites of PLoS Medicine at http://www.plosmedicine.org/, Annals of Internal Medicine at http://www.annals.org/, and Epidemiology at http://www.epidem.com/). Information on the STROBE Initiative is available at www.strobe-statement.org.
