# Supplementary material for: The relationship between violence history in patients with severe mental disorders and child abuse of their children
Source: PeerJ. 2026 Apr 7;14:e21028. doi: 10.7717/peerj.21028 (PMC13068010; doi:10.7717/peerj.21028)
Supplement: Supplemental Information 2 [file peerj-14-21028-s002.docx]

# Risk Behavior Assessment scale

# Risk Behavior Assessment scale is derived from the "Management and Treatment Work Specifications for Severe Mental Disorders" released by the National Health Commission of the People's Republic of China in 2018. detail as follows：

Level 0: no behavior in the following 1–5 levels.

Level 1: Verbal threats, shouting but not destruction of property.

Level 2: Patients destroy property in their homes, but the violence was confined to their homes and could be stopped by persuasion.

Level 3: Regardless of the occasion, obvious behavior of destroy property persisted and persuasion cannot stop the violence.

Level 4: The patient continued to hurt others and destroy property, regardless of the occasion, and could not stop violent behavior (including self-harm and suicide) after persuasion.

Level 5: Use of dangerous instruments to commit acts of violence against people in their homes or public places or commit acts of violence, such as arson.
