# Supplementary material for: The relationship between violence history in patients with severe mental disorders and child abuse of their children
Source: PeerJ. 2026 Apr 7;14:e21028. doi: 10.7717/peerj.21028 (PMC13068010; doi:10.7717/peerj.21028)
Supplement: Supplemental Information 3 [file peerj-14-21028-s003.doc]

Did family member experience...

1.being pushed, grabbed, slapped, or having something thrown at him/her?

2.being hit so hard that he/she had marks or bruises or was injured?

3.being threatened or hurt with a knife or gun?

4.being yelled, screamed, or cursed at?

5.being insulted or humiliated?

Did a parent or family member...

6.swear at you, insult you, or put you down?

7.threaten to hurt you physically?

8.push, grab, slap, or throw something at you?

9.hit you so hard that you had marks or bruises or were injured?
