# Supplementary material for: The relationship between violence history in patients with severe mental disorders and child abuse of their children
Source: PeerJ. 2026 Apr 7;14:e21028. doi: 10.7717/peerj.21028 (PMC13068010; doi:10.7717/peerj.21028)
Supplement: Supplemental Information 5 [file peerj-14-21028-s005.docx]

| variable | category |
| --- | --- |
| abuse or ridicule | 0=no 1=yes |
| being threated | 0=no 1=yes |
| being pushed, grabbed or thrown | 0=no 1=yes |
| severe beatings | 0=no 1=yes |
| At least one type of abuse | 0=zero 1=more than one |
| Multiple types of abuse | 0=zero 1=one 2=more than two |
| Gender of children | 1=boy 2=girl |
| Age of children | 1=9~11 2=12~14 3=15~17 |
| Gender of SMD | 1=male 2=female |
| Age of SMD | 1= ~40 2=40-50 3=50~ |
| Currently attending school | 1=yes 2=no |
| Residence place | 1=Rural 2=Urban |
| Household poverty status | 0=Non-poverty 1=Poverty |
| Individuals with mental illness | 0=one 1=two |
| Presence of physical health problems in family members | 0=no 1=yes |
| Relapse of mental illness in family members | 0=no 1=yes |
| Duration of SMD （years） | 1=≤5 2=＞5 |
| Type of SMD | 1=Schizophrenia 2=Others |
| Education of SMD | 1=Illiterate or primary school, 2=Junior middle school,3=Senior high school |
| Hospitalization times of SMD | 0=once 1=twice 2=more than twice |
| Violence history of SMD | 0=no 1=yes |
